# Effects of Aerobic Exercises Combined With Music Therapy on Fatigue and Quality of Life in Females With Thyroidectomy Following Thyroid Cancer: An Experimental Study

**DOI:** 10.7759/cureus.79071

**Published:** 2025-02-15

**Authors:** Akshaya Saklecha, Moh'd Irshad Qureshi, Raghumahanti Raghuveer, Pallavi Harjpal, Shubhangi Patil

**Affiliations:** 1 Department of Neurophysiotherapy, Ravi Nair Physiotherapy College, Datta Meghe Institute of Higher Education and Research, Wardha, IND

**Keywords:** aerobic exercises, fatigue, fatigue assessment scale, music therapy, post thyroidectomy, sf-36 questionnaire, thyroid cancer

## Abstract

Background

Thyroid cancer is a type of cancer that starts in the thyroid gland. Thyroid cancer is characterized by the abnormal and uncontrolled growth of cells within the thyroid gland. In recent years, the incidence of thyroid cancer has been rising globally, with a significant increase observed in India. It is more prevalent among females, who are three times more likely to be diagnosed with thyroid cancer than males. According to recent statistics, thyroid cancer constitutes approximately 4%-6% of all cancers among Indian women, with an estimated incidence rate of 8.7 per 100,000 women. Thyroidectomy is a technique performed to manage thyroid cancer. Females who undergo thyroidectomy and receive insufficient levothyroxine supplementation are more prone to experience problems such as fatigue, which can significantly affect their quality of life (QoL). Fatigue is a prevalent and discomforting symptom among oncology patients, though its prevalence can vary depending on cancer type, stage, and treatment. Exercise is becoming widely recognized as a management strategy for fatigue and for enhancing QoL. This study aims to evaluate the effects of aerobic exercise and music therapy on fatigue and QoL in females who have undergone thyroidectomy due to thyroid cancer. Fatigue will be measured using the Fatigue Assessment Scale (FAS), while QoL will be assessed through the RAND SF-36 Questionnaire. The study seeks to determine how these combined interventions may help improve both physical and mental well-being in this population.

Method

The study was conducted in the physiotherapy outpatient department of Acharya Vinoba Bhave Rural Hospital over a duration of six months, using an experimental interventional design. A total of 30 female participants, all of whom have undergone thyroidectomy due to thyroid cancer, were selected through a simple random sampling method with allocation based on a computer-generated list. Female participants will ensure completion of the six-week session. As interventions, both aerobic exercises and music therapy were combined. The RAND SF-36 Questionnaire and FAS were utilized to examine QoL and fatigue levels before and after treatment. The student-paired t-test was applied to compare pre- and post-treatment outcomes.

Result

Following six weeks of interventions, the fatigue symptom was reduced, resulting in a considerable enhancement in QoL. Post-intervention the FAS (t=13.37, p=0.0001) and RAND SF-36 (t=14.09, p=0.0001) scores improved more significantly. The level of significance was found to be p<0.0001.

Conclusion

Based on the findings of this study, it can be concluded that the combination of aerobic exercises and music therapy has a positive impact on the QoL of females who have undergone thyroidectomy. Additionally, these interventions were found to effectively reduce fatigue symptoms in this population. These results underscore the potential benefits of integrating aerobic exercises and music therapy into post-thyroidectomy care strategies for enhancing overall well-being.

## Introduction

Thyroid cancer is an endocrine cancer that mostly affects women and is caused by uncontrolled growth and transformation of healthy thyroid cells, which leads to the development of a tumor in the thyroid gland [1]. It is characterized by the abnormal growth of thyroid gland cells. Thyroid cancer may not show any signs or symptoms initially. However, when it progresses, it can develop signs and symptoms such as enlargement in the neck, voice changes, and swallowing difficulties. Thyroid cancer, the fifth most frequently occurring cancer globally, has seen a significant increase in prevalence in India, particularly among younger individuals. It affects more females than males and is being detected at a younger age [2]. An estimated 70% of all reported cases are found in people below the age of 55, with only 2% occurring in children and teens [3]. The prognosis for this cancer varies significantly by histologic subtype; while more than 90% of patients with ductal and lobular carcinoma experience long-term survival, those with anaplastic carcinoma face a much more proper outlook, often resulting in death. As a result, improving life quality is a top concern, particularly for young cancer survivors.

Medical management with surgery, radiation, and other treatments is the primary approach for managing thyroid cancer. Physiotherapy strategies play a valuable role in improving quality of life (QoL) and functional outcomes for survivors, particularly after treatments that may impact physical function. While these treatments hold the potential to cure some illnesses and lengthen the lives of people with others, they are linked to several other physical and mental side effects [4]. Symptoms, such as fatigue, nausea, vomiting, hair loss, appetite loss, constipation, difficulty sleeping, discomfort, weight changes, and amenorrhea, can be linked to various thyroid conditions, including hypothyroidism and thyroid cancer. While these symptoms are commonly recognized in hypothyroidism, they may also manifest in thyroid cancer patients due to the disease itself or as side effects of treatment. Thyroidectomy is a commonly recommended treatment for differentiated thyroid cancer; however, in advanced disease, particularly stage IV, where the cancer is deemed unresectable, treatment options may include tyrosine kinase inhibitors as a primary approach [5]. Females who have undergone thyroidectomy may experience a negative impact on their QoL, often manifesting as fatigue and other problems.

Fatigue has a profound influence on the performance of life of cancer patients, resulting in poor QoL, and it is the most prevalent adverse effect of thyroid cancer management [5]. It is estimated that fatigue will affect between 70% and 100% of cancer patients. As per the National Comprehensive Cancer Network (NCCN), fatigue is described as an unpleasant, persistent, subjective experience of physical, emotional, and/or cognitive fatigue associated with illness or cancer management that is not equivalent to current events and significantly interferes with typical functioning [6]. Exercise is one method suggested for reducing fatigue.

The Oncology Nursing Society and NCCN both promote exercise as a way to minimize fatigue in oncology patients who are during, post, and end-of-life treatment. According to the NCCN clinical practice guidelines, exercise programs for cancer patients should start gradually, increase slowly in intensity, and be customized to the needs of patients. Likewise, the American College of Sports Medicine (ACSM) advises all cancer patients to follow exercise guidelines, not just to reduce fatigue but also to enhance QoL [7]. Aerobic exercise was demonstrated to enhance the physical activity of patients by reducing fatigue due to cancer. As per ACSM, aerobic exercise was defined as the continuous rhythmic contraction and relaxation of major groups of muscles over an extended time [8].

On the other hand, music therapy for cancer survivors is also the best treatment option for promoting physical health and thus improving QoL. Music therapy is a behavioral science that employs music therapies systematically to regain, maintain, and improve emotional and physiological health and well-being [9]. It was also effective in providing relaxation and relieving fatigue in cancer patients [10]. Music had a good effect on health and well-being by lowering fatigue. When music and exercise are combined, the physical and psychological advantages are great. Listening to music while exercising has an ergogenic impact, which means it improves exercise performance [11]. Aerobic exercise, according to other research, can optimize neuroendocrine function by increasing endorphin and serotonin levels [12], alleviate fatigue, improve functions of the body, and promote healthy lifestyles, whereas music therapy also provides sedative effects, thus relieving the patient's symptoms and improving QoL [13]. This study aimed to investigate the impact of aerobic exercises and music therapy on fatigue and QoL in females who underwent thyroidectomy after thyroid cancer.

## Materials and methods

Study design and setting

The study was conducted after obtaining ethical clearance from the Institutional Ethics Committee of Datta Meghe Institute of Medical Sciences (approved number: DMIMS(DU)/IEC/2021/492). Subjects were selected for the research. The study's research design is an interventional study, whereas the study design is an experimental study. The study was conducted in the physiotherapy outpatient department of Acharya Vinoba Bhave Rural Hospital over a duration of six months, allowing for participant recruitment, intervention implementation, data collection, and analysis.

Sample size calculation

To determine the sample size for this study, the following formula was used [14]: n = Zα/22.P. (1-P)/d2.

In this formula, Zα/2 represents the level of significance set at 5%, corresponding to a 95% confidence interval, which equals 1.96. The variable P denotes the incidence of thyroid cancer, calculated as 3.7%, or 0.037. The term d refers to the desired margin of error, which has been set at 7%, or 0.07. Substituting the values into the formula:

n = 1.96 2 × 0.0037× (1-0.037)/0.072 = 30 patients needed in the study.

This calculation indicated that 30 patients would be required for the study. The study was designed with an 80% power and a 5% level of significance. A simple random sampling technique was employed, and data were analyzed using SPSS version 27.0 (IBM Corp., Armonk, NY). The chi-square test was utilized for statistical analysis.

Participant selection

This study enrolled 30 females who underwent either total or hemithyroidectomy following a diagnosis of thyroid cancer through a simple random sampling method. The staging of thyroid cancer among participants ranged from stage I to stage IV, as determined by the AJCC TNM classification system. The allocation method was based on a computer-generated list. Participants were chosen based on inclusion and exclusion criteria. The research objectives and approaches were communicated to the participants, who then signed written patient consent forms. The participants were selected based on the inclusion and exclusion criteria shown in Table 1.

**Table 1:** The inclusion and exclusion criteria of the study

Inclusion criteria	
1.	Only female participants
2.	Participants aged between twenty to fifty-five years old
3.	Participants who had undergone thyroidectomy following a diagnosis of thyroid cancer
4.	Participants experiencing cancer-related fatigue and a low quality of life following a thyroidectomy.
Exclusion criteria	
1.	Participants with a physician-identified cardiovascular disease.
2.	Participants who are currently enrolled in another research study.
3.	Participants with a long-term, progressive medical problem.
4.	Participants who are currently pregnant

Treatment procedure 

Participants underwent a rehabilitation program consisting of aerobic exercises combined with music therapy. The intervention was conducted four times a week for six weeks, with each session lasting 40 minutes, structured as follows.

The rehabilitation program began with a 10-minute warm-up phase designed to gradually prepare participants for the upcoming aerobic activity. During this time, participants engaged in spot marching, a series of neck stretches, and upper-extremity range of motion exercises. The intensity of these movements was kept at a low level, emphasizing gentle motions that gently warmed up the muscles without causing strain or discomfort. This warm-up routine was consistently performed at the beginning of each rehabilitation session.

Following the warm-up, participants transitioned into the main component of the program: 20 minutes of aerobic exercise combined with personalized music therapy. During this phase, Participants gathered in a quiet and comfortable environment to relax their minds and bodies in the patient service center. Music therapy was administered and participants walked at a moderate intensity. The integration of music therapy, with participants listening to songs tailored to their individual preferences, aimed to enhance motivation, mood, and overall enjoyment during the exercise session. Participants used headphones for the treatment, with the volume set at 30-50 dB. This aerobic exercise with music therapy was conducted four times per week throughout the six-week intervention period.

To conclude each rehabilitation session, participants engaged in a 10-minute cool-down phase. This phase focused on promoting relaxation and flexibility, with participants performing breathing exercises, neck stretches, and stretches targeting the hamstrings and calves. The intensity of these cool-down activities was low, emphasizing gentle movements and deep breathing to help the body transition from the aerobic exercise back to a resting state. This cool-down routine was consistently performed at the end of each rehabilitation session.

Patients were required to attend treatment sessions four days a week for six weeks. Participants were asked to respond to the Rand SF-36 and FAS pre- and post-treatment. Fatigue levels were assessed using the FAS, while QoL was evaluated using the Rand SF-36 questionnaire.

Outcome measures

Fatigue Assessment Scale (FAS) is a self-answerable questionnaire that was used to evaluate fatigue. It is an effective tool for a large number of cancer patients and has good validity and reliability with Cronbach's alpha 0.90. This scale is graded on a Likert scale, which is a 5-point scale that ranges between 1 and 5 on how patients usually feel, where 1 denotes never and 5 denotes always. Total scores can vary from 10 to 50, with 10 representing the least and 50 indicating the greatest fatigue [15]. Rand SF-36 Questionnaire is currently the most widely used health-related quality-of-life measure and it was used to determine QoL of the patients. It has a total of 36 items and is divided into eight main domains. The eight domains of health encompass physical functioning, limitations due to physical issues, role restrictions due to emotional disorders, social functioning, emotional well-being, fatigue, general health perceptions, and pain. The scale was shown to be reliable for people with cancer [16].

Statistical analysis

The statistical analysis of the data was conducted using SPSS version 27.0 software. Both descriptive and inferential statistics were employed to assess the outcomes of the intervention. Descriptive statistics were calculated to summarize the demographic and baseline characteristics of the participants, as well as the pre- and post-intervention scores for QoL and fatigue levels. Before conducting inferential statistics, we assessed the normality of the data distribution using the Shapiro-Wilk test. Based on the results, the p-value was greater than 0.05 indicating that the data were normally distributed, allowing us to use parametric tests. For inferential analysis, we utilized Student's paired t-test to compare pre- and post-intervention scores for both QoL and fatigue levels. The significance level is considered at p<0.05.

## Results

In this study, 30 patients who met the eligibility criteria were enrolled to investigate the effect of aerobic exercises combined with music therapy on fatigue and QoL post-thyroidectomy. The Statistical Analysis was conducted utilizing the SPSS 27.0 software. Descriptive and inferential statistics were calculated for all variables using the Student’s paired t-test. If the results showed a p-value (a measure of statistical significance) less than 0.05, it was considered significant and marked as “S.” If the p-value was more than 0.05, it was considered not significant and marked as “NS.”

Patient demographics

A total of 30 female patients participated in the study. The majority of the participants were within the 31-40 years age group, comprising 12 patients (40%). This was followed by the 21-30 years age group, which included 11 patients (36.67%). The remaining seven participants (23.33%) were aged between 41 and 50 years. The mean age of the study population was 34.60 years, with a standard deviation of 6.91 years, indicating a moderate variation in age among the participants. The age range of the participants was from 25 to 49 years. Table 2 depicts the patient age distribution.

**Table 2:** Patient age distribution (years)

Age group (years)	No of patients	Percentage
21-30	11	36.67
31-40	12	40
41-50	7	23.33
Total	30	100
Mean±SD	34.60 ± 6.91(25-49 years)	

Comparison of outcomes: FAS and RAND SF-36 scores

The effect of the intervention on fatigue and QoL was analyzed using descriptive statistics and Student's paired t-test, with statistical analysis performed using SPSS version 27.0. A p-value of less than 0.05 was considered significant. Table 3 presents a detailed comparison of the fatigue assessment scale (FAS) and RAND SF-36 scores pre- and post-treatment.

**Table 3:** Comparison of FAS and RAND SF-36 scores pre- and post-treatment p=0.0001 implies statistically significant results marked as “S.” FAS - Fatigue assessment scale,

Outcome	Treatment	Mean	Standard deviation	Standard error mean	Mean difference	t- value	P-value
FAS	Pre-treatment	34.00	5.91	1.07	14.60±5.98	13.37	P=0.0001, S
	Post-treatment	19.40	4.58	0.83			
RAND SF-36	Pre-treatment	40.27	15.43	2.81	35.55±13.81	14.09	P=0.0001, S
	Post-treatment	75.83	15.41	2.81			

Fatigue assessment scale

The mean FAS score at pre-treatment was 34 ± 5.91, indicating a moderate level of fatigue among the participants prior to the intervention. After the intervention, the mean FAS score significantly decreased to 19.40 ± 4.58. The mean difference between pre- and post-treatment scores was 14.60 ± 5.98. The analysis using Student’s paired t-test revealed a statistically significant difference (t = 13.37, p = 0.0001), resulting in a significant reduction in fatigue levels following the intervention. This indicates a substantial improvement in fatigue symptoms after the combined aerobic exercise and music therapy.

RAND SF- 36

The mean RAND SF-36 score, which reflects the QoL, was 40.27 ± 15.43 at pre-treatment, signifying a low QoL in participants before the intervention. Post-treatment, the mean RAND SF-36 score markedly improved to 75.83 ± 15.41, indicating a significant enhancement in the participants' QoL. The mean difference between pre- and post-treatment scores was 35.55 ± 13.81. This difference showed a statistically significant improvement in the RAND SF-36 scores following the intervention according to Student’s paired t-test (t = 14.09, p = 0.0001). This suggests that the combination of aerobic exercise and music therapy had a profound positive effect on enhancing the QoL of the study participants.

These results underscore the effectiveness of the combined aerobic exercise and music therapy intervention in significantly reducing fatigue and enhancing the QoL for post-thyroidectomy patients. The statistically significant improvements observed in both FAS and RAND SF-36 scores suggest that this therapeutic approach could be a valuable addition to the post-operative care of individuals recovering from thyroidectomy.

## Discussion

On the basis of the result of our study, we found that females in the age group of 25 to 49 years' experience fatigue and poor QoL as a result of thyroidectomy following thyroid cancer. In this study, 30 females were included who underwent thyroidectomy. Fatigue related to cancer and QoL were analyzed as outcome measures by using the FAS and RAND SF-36 questionnaires. After completion of the exercise program, patients reported lower fatigue and an enhancement in QoL. There were visible changes in pre- and post-FAS scoring as well as in RAND SF-36 scoring. The findings of this study revealed that after the completion of six weeks of aerobic exercises along with music therapy, there was a remarkable reduction in fatigue related to cancer and enhancement in QoL, and both comparison values were significant (p = 0.0001). According to the findings of the research, the intervention had a positive impact on fatigue, thus enhancing QoL. The study found that aerobic exercises and music therapy significantly improved the QoL and fatigue in females undergoing thyroidectomy.

Thyroid cancer is one of the most common malignancies in females, manifested by a tumor in the thyroid gland. The thyroid gland synthesizes thyroid hormones by absorbing iodine from the bloodstream, where these hormones play a crucial role in regulating cellular metabolism, energy production, and overall metabolic rate. Thyroid cancer cells often exhibit poor iodine uptake due to functional alterations; however, iodine remains a critical treatment option and follow-up tool, particularly in the context of radioactive iodine therapy, which is used to target and destroy residual cancerous tissue. Changes in iodine metabolism caused by defective thyroglobulin synthesis contribute to the development of thyroid cells. On the benefits of daily aerobic exercises in the early post-treatment period to reduce fatigue and improve QoL, a physiotherapist is a prime role model in educating cancer survivors. As it enhances the overall general health of patients and makes a healthy lifestyle. Aerobic exercise has a significant impact on one's mood and, as a result, improves one's QoL [17].

Research conducted by Maki et al. on 292 thyroid cancer patients said that fatigue was common among cancer survivors and was linked to a low QoL. This present study was supported by our study findings. This study assesses fatigue and QOL; they used the SF-36 version and the cancer fatigue scale. This study concluded that cancer patients show fatigue and poor QoL [18]. Therefore, as per this study, after thyroid cancer, there were chances to develop fatigue and poor QOL. The finding of this study was in favor of the study performed by Petruzzello et al., which disclosed that aerobic exercise is a great tool to reduce anxiety, and it continues to support the research results of the overview by showing that anxiety and fatigue levels decreased after aerobic exercise training. The results of this study concluded that for reducing anxiety and fatigue, aerobic exercise with music therapy was beneficial [19].

Music therapy is involved in many studies; researchers have shown that music is a relaxation tool. Music had a positive influence on health and well-being by lowering fatigue. Musical therapy has been used to help cancer patients relax. The findings of this current study were given by Grocke et al., who evaluated the effect of music, which is anxiolytic. The implementation of music therapy in oncology patients had a great impact on music, which acts as a preventive, curative, and palliative cancer care.

Many studies show that, despite the fact that music therapy has no direct relationship to the condition, it has a significant impact on mood and healing power, as well as a change in how the patient attempts to cope with his thoughts about the disease. Music therapy emphasizes and concentrates on both the physiological and psychological demands as a result of this disease, as well as the treatment's aftereffects and adverse effects.

According to our research, we found that the patients who have undergone thyroidectomy have fatigue and reduced QoL. Physical inactivity for a long duration after surgery in females was the reason for reduced QoL. The study done by Dimeo concluded that lowering fatigue and enhancing psychologic distress in patients following chemotherapy. Aerobic exercises had a positive effect [20].

The limitations of the study are the small sample size of 30 participants and the lack of follow-up for the patients. The study was conducted over a short duration, which suggests that a similar study needs to be conducted over a longer period to thoroughly evaluate the effectiveness of interventions in females after thyroidectomy. While this study specifically targeted fatigue related to cancer and QoL, future research could also focus on menstrual irregularities and anxiety in females after thyroidectomy.

## Conclusions

The study findings highlight the significant impact of aerobic exercises combined with music therapy on reducing fatigue and improving the QoL in females post-thyroidectomy. This research underscores the importance of incorporating both aerobic exercises and music therapy into the management of fatigue and overall well-being. The results suggest that a six-week exercise protocol involving these interventions not only alleviates cancer-related fatigue but also enhances the overall QoL for individuals undergoing post-thyroidectomy recovery. This study sheds light on the potential benefits of combining physical activity with music therapy in improving health outcomes and underscores the need for further exploration and integration of these interventions in clinical practice.

## Appendices

**Table 4:** Fatigue assessment scale (FAS)

Sr no.	Questions	Never	Sometimes	Regularly	Often	Always
1	I am bothered by fatigue	1	2	3	4	5
2	I get tired very quickly	1	2	3	4	5
3	I don't do much during the day	1	2	3	4	5
4	I have enough energy for everyday life	5	4	3	2	1
5	Physically, I feel exhausted	1	2	3	4	5
6	I have problems to start things	1	2	3	4	5
7	I have problems to think clearly	1	2	3	4	5
8	I feel no desire to do anything	1	2	3	4	5
9	Mentally, I feel exhausted	1	2	3	4	5
10	When I am doing something, I can concentrate quite well	5	4	3	2	1
